# LncRNA SLCO4A1-AS1 modulates colon cancer stem cell properties by binding to miR-150-3p and positively regulating SLCO4A1

**DOI:** 10.1038/s41374-021-00577-7

**Published:** 2021-05-06

**Authors:** Kun Wu, Ting Xu, Xudong Song, Jie Shen, Shutao Zheng, Li Zhang, Guoquan Tao, Baofei Jiang

**Affiliations:** 1 Department of Gastrointestinal Surgery, The Affiliated Huai’an No.1 People’s Hospital of Nanjing Medical University, Huai’an, 223300 PR China; 2 Department of Hematology, The Affiliated Huai’an No.1 People’s Hospital of Nanjing Medical University, Huai’an, 223300 PR China; 3 The Second Clinical College, Nanjing University of Chinese Medicine, Nanjing, 210023 PR China; 4 Clinical Medical Research Institute, The First Affiliated Hospital of Xinjiang Medical University, Urumqi, 830011 PR China; 5 VIP Medicine, The First Affiliated Hospital of Xinjiang Medical University, Urumqi, 830054 PR China

**Keywords:** Cancer, Cell growth

## Abstract

Long non-coding RNAs (lncRNAs) play important roles in a range of different human cancers. However, the role of lncRNA solute carrier organic anion transporter family member 4A1-AS1 (SLCO4A1-AS1) in colon cancer remains enigmatic. Hence, we aimed to explore the specific role of SLCO4A1-AS1 in colon cancer stem cells. Colon cancer-related differentially expressed lncRNA and mRNA were screened using microarray-based analysis, and the expression of SLCO4A1-AS1 and SLCO4A1 in colon cancer tissues was determined using reverse transcription quantitative polymerase chain reaction and western blot analysis. The interaction among SLCO4A1-AS1, microRNA-150-3p (miR-150-3p) and SLCO4A1 was verified using dual-luciferase reporter assay, RNA immunoprecipitation and RNA pull-down. Moreover, SLCO4A1-AS1, miR-150-3p and/or SLCO4A1 were overexpressed or depleted in colon cancer cells to detect their effects on migration, invasion, sphere formation, apoptosis and tumorigenesis abilities of colon cancer stem CD133^+^CD44^+^ cells using both in vitro and in vivo assays. SLCO4A1-AS1 and SLCO4A1 were screened as the differentially expressed lncRNA and mRNA in colon cancer tissues. SLCO4A1-AS1 was confirmed to competitively bind to miR-150-3p to elevate SLCO4A1 expression. Moreover, knockdown of SLCO4A1-AS1 decreased SLCO4A1 expression, thus inhibiting cell migration, invasion, sphere formation, and tumorigenesis abilities and enhancing the apoptosis of CD133^+^CD44^+^ cells. Collectively, these findings provide evidence demonstrating that depleting SLCO4A1-AS1 competitively binds to miR-150-3p, which downregulates SLCO4A1 expression, thus hindering colon cancer progression.

## Abstract (web summary)

This study reports that long non-coding RNA lncRNA SLCO4A1-AS1 regulates the characteristics of colon cancer stem cells. Moreover, SLCO4A1 targets miR-150-3p. LncRNA SLCO4A1-AS1 can be used as ceRNA to adsorb miR-150-3p and thus affect the expression of SLCO4A1. Mechanistically, miR-150-3p can downregulate SLCO4A1, thereby inhibiting migration, invasion, spheroidization and tumor formation of colon cancer stem cells. This study lays a theoretical foundation for in-depth understanding of the pathogenesis of colon cancer and points to new therapeutic targets.

## Introduction

Colon cancer is the third most common cancer in men and the second in women, imposing high disease burden on public health [1]. The prognosis and treatment of colon cancer are mainly monitored by histologic tumor staging [2]. Cancer stem cells, also known as tumor-initiating cells, are self-renewing and aggressive cells that often lead to the development and occurrence of numerous types of cancers including colon cancer [3]. Therefore, targeting cancer stem cells directly could help to enhance the diagnosis and therapy of colon cancer.

Recently, long non-coding RNAs (lncRNAs) and microRNAs (miRNAs) have been shown to play important roles in colorectal cancer, and thus can be used as diagnostic markers as well as therapeutic targets [4, 5]. LncRNAs are often dysregulated in cancers, with a specific example being solute carrier organic anion transporter family member 4A1-AS1 (SLCO4A1-AS1) [6]. SLCO4A1-AS1 had been shown to be highly expressed in bladder cancer, while the downregulation of SLCO4A1-AS1 inhibited the proliferation, invasion and migration abilities of bladder cancer cells [7]. Similarly, SLCO4A1-AS1-202 was reported to exert an important role in the initiation and proliferation of colorectal cancer cells [8], and as a result SLCO4A1-AS1-202 is considered a prognosis marker of colorectal cancer [9]. miRNAs also play a key role in the occurrence and development of cancers [10]. In the present study, we predicted that SLCO4A1 was a target gene of miR-150-3p. Interestingly, it had previously been shown that miR-150-3p inhibits the development of several types of cancers [11]. For example, miR-150 exerts an inhibitory effect on cell proliferation, while accelerates cellular apoptosis in colorectal cancer [12]. However, a direct link between SLCO4A1-AS1 and miR-150-3p has not been identified, while the mechanism underlying miR-150-3p in colon cancer progression remains unclear.

In this study, bioinformatics analysis demonstrated that SLCO4A1-AS1 could bind to miR-150-3p, and SLCO4A1 might be a target gene of miR-150-3p. Our study aims to characterize the role of SLCO4A1-AS1 in the regulation of colon cancer stem cells and we have shown that SLCO4A1-AS1 affects the progression of colon cancer stem cells by interacting with miR-150-3p and SLCO4A1, which contributes to finding more targets for the treatment of colon cancer.

## Materials and methods

### Microarray-based analysis

Differentially expressed lncRNAs and mRNAs were obtained from the Gene Expression Profiling Interactive Analysis (GEPIA) (http://gepia.cancer-pku.cn/) website, and the correlation between lncRNA and mRNA was predicted *via* the ChIPBase v2.0 website (http://rna.sysu.edu.cn/chipbase/index.php).

### Cell culture

Human colon cancer cell HCT116 and colon epithelial cell NCM460 were purchased from American type culture collection (ATCC) (Manassas, VA, USA) and cultured in Dulbecco’s Modified Eagle’s Medium (DMEM) containing 10% fetal bovine serum (FBS) in an incubator at 37 °C with 5% CO_2_. When the confluence reached 90%, the cells were trypsinized (0.25%) and sub-cultured. Cells in the logarithmic growth phase were used for the experiment. HCT116 cells expressing CD133^+^CD44^+^ were sorted by flow cytometer for subsequent analysis. Expression of SLCO4A1 in colon cancer cell HCT116, CD133^+^CD44^+^ cells and colon epithelial cell NCM460 was determined by reverse transcription quantitative polymerase chain reaction (RT-qPCR).

### Cell treatment

The cells were transfected with TurboFect transfection reagent (Thermo Scientific) using cationic polymer technology. Briefly, HCT116 cells were seeded in 24-well plates at a density of 6 × 10^4^ cells/well and cultured in FBS-free DMEM. The purified pEGFP-N1 vector (~1 g) was pre-incubated with 4 μL reagent at a final volume of 25 μL and incubated at room temperature for 20 min to form a DNA/TurboFect complex. The complex was then added to each well containing serum-free medium. After incubation at 37 °C for 6 h, the medium was renewed with RPMI containing 5% FBS. Transfection efficiency was examined under a fluorescence microscope (Envert Fluorescent Ceti, Korea) and quantified by a FACS Calibur flow cytometer (Partec, Germany) 24 h after transfection. Flow cytometry settings were adjusted to distinguish between transfected and untransfected cells. Windows FloMax software package was used for data analysis. In addition, in order to treat with both chemical reagents and mechanical loading, cells cultured on silicon membranes were loaded through various mechanical bioreactors prior to transfection. Then, plasmid DNA in the presence or absence of TurboFect was added to the medium and the cells were cultured in a 37 °C, 5% CO_2_ incubator. Cells were grouped into the vector SLCO4A1 group (transfected with SLCO4A1 plasmid overexpression vector 8 μL), the vector NC group (transfected with empty plasmid overexpression vector group 8 μL), si-SLCO4A1 group (transfected with SLCO4A1 silencing vector 8 μL), si-NC group (transfected with empty plasmid vector 8uL), mimic NC group (transfected with empty plasmid vector 8 μL), miR-150-3p mimic group (transfected with miR-150-3p overexpression vector 8 μL), miR-150-3p inhibitor (transfected with miR-150-3p silencing vector), inhibitor NC (transfected with empty plasmid vector), miR-150-3p mimic + vector NC (transfected with miR-150-3p overexpression plasmid vector and empty plasmid vector), miR-150-3p mimic + vector SLCO4A1 (transfected with miR-150-3p overexpression plasmid vector and SLCO4A1 group overexpression plasmid vector), oe-NC + miR-150-3p mimic (transfected with SLCO4A1-AS1 overexpression plasmid vector and empty plasmid vector), oe-SLCO4A1-AS1 + miR-150-3p mimic (transfected with SLCO4A1-AS1 overexpression plasmid vector and miR-150-3 overexpression vector).

### Scratch test

The cells in logarithmic growth phase were collected 48 h after transfection, seeded into six-well plates at 1 × 10^6^ cells per well and cultured in an incubator at 37 °C with 5% CO_2_ until the cell confluence was about 95%. Afterwards, a 20 μL micropipette was used to make vertical linear scratches in a six-well plate, and D-hanks solution was applied to remove the falling cells. After that, the cells continued to be cultured in serum-free media. Cells were then imaged 0 and 48 h after scratching. Three visual fields (200×) were randomly selected for image acquisition under a phase contrast microscope. The differences in scratch healing among different groups were compared and the healing rate interpreted as the cell migration ability.

### Transwell assay

Matrigel (354230, Shanghai qcbio Science & Technologies co., Ltd., Shanghai, China) was melted overnight at 4 °C, and diluted at a ratio of 1:3 with serum-free 1640 medium. The apical chamber of each Transwell chamber was coated with diluted Matrigel. Next, 48 h transfection, HepG2 cells from all groups were collected and seeded in the apical chamber of Transwell chamber (Corning Incorporated, Corning, NY, USA), and 0.5 mL 1640 medium containing 10% FBS was added to the basolateral chamber of the 24-well plate. The cells were cultured in an incubator at 37 °C with 5% CO_2_ for 48 h. The cells not penetrating the membrane in the Transwell chamber were gently removed, and the cells were fixed in 95% ethanol for 15–20 min, stained with methylium violaceum for 10 min, washed, and observed under a high-inverted microscope. Finally, five visual fields were selected to calculate the average number of cells in each group. The number of cells passing through Matrigel was used as an indicator for judging the invasive ability of the cells.

### Suspension sphere formation assay

Single-cell suspension was prepared in serum-free stem cell medium and then seeded into the 24-well ultra-low adhesion culture plate at a density of 1000 cells per well. After 5-day culture, the formation of tumor spheres was observed under a Nikon Eclipse TE2000-S microscope.

### Flow cytometry

After 48 h of transfection, cells were collected. Then cell apoptosis was detected using an Annexin V-fluorescein isothiocyanate (FITC)/propidium iodide (PI) apoptosis detection kit (CA1020, Beijing Solarbio Science & Technology Co., Ltd., Beijing, China). The cells were washed using binding buffer and incubated with Annexin V-FITC diluted with binding buffer at a ratio of 1:40 and incubated in the dark at room temperature for 30 min. Then the cells were incubated with PI, diluted with binding buffer at a ratio of 1:40 for 15 min. Cell apoptosis was detected by flow cytometry.

### Dual-luciferase reporter assay

The bioinformatics prediction website (https://cm.jefferson.edu/rna22/) was used to identify binding sites among miR-150-3p, SLCO4A1-AS1 and SLCO4A1. The full length of SLCO4A1-AS1 and the 3′ untranslated region (UTR) of SLCO4A1 were cloned into the pmirGLO (E1330, Promega Corp., Madison, WI, USA) Luciferase vector respectively to construct pSLCO4A1-AS1 wild type and pSLCO4A1 wild type. pSLCO4A1-AS1 mutant type and pSLCO4A1 mutant type in which the binding sites of miR-150-3p were mutated were also constructed separately. pRL-TK plasmid (E2241, Promega Corp., Madison, WI, USA) expressing renilla luciferase was taken as the internal reference. miR-150-3p mimic and miR-150-3p mimic NC were co-transfected with luciferase reporter plasmids respectively into CD133^+^CD44^+^ cells (CRL-1415, ATCC, Manassas, VA, USA). The Dual-Luciferase Reporter Gene Assay Kit (GM-040502A, Shanghai qcbio Science & Technologies Co., Ltd., Shanghai, China) was employed to detect luciferase activity at 560 nm (firefly relative luminescence units (RLU)) and 465 nm (renilla RLU), and the ratio of firefly RLU/renilla RLU was calculated.

### RT-qPCR

CD133^+^CD44^+^ cells in logarithmic growth phase were collected 48 h after transfection. Total RNA was extracted under the instruction of miRNeasy Mini Kit (QIAGEN, Hilden, Germany). A total of five RNA sample was taken and diluted with RNAase free water. Absorption at 260 and 280 nm was measured using a UV spectrophotometer, and the concentration and purity of RNA were determined. Then 12 μL RNAase free water, 2 μL ODT and 3 μL RNA sample were added into each 200 μL RNAase free centrifuge tube. After heated at 70 °C for 5 min, the sample was immediately cooled on ice water for 2 min. Then 1 μL dNTP, 1 μL guanidine isothiocyanate, 5 μL 5 × reverse transcription buffer, 1 μL reverse transcriptase MMLV were added, and then the mixture was gently mixed with a pipette, and then heated at 37 °C for 90 min. The reaction was terminated by heating at 70 °C for 5 min and stored in an ice box for further experiments. The target gene and reference gene were amplified by fluorescence quantitative PCR (ABI 7500, Applied Biosystems, USA). The PCR reaction system consisted of 25 μL 10 × PCR buffer, 2.5 μL 25 mmol/L MgCl 2, 1.5 μL 10 mmol/L dNTP, 0.5 μL 10 mmol/L primer, 1 μL 1 nmol/L probe, 0.25 μL Taq, 2.5 μL cDNA and 15 μL RNAase free water. The reaction conditions were as follows: denaturation at 94 °C for 5 min, denaturation at 94 °C for 30 s, denaturation at 58 °C for 45 s, denaturation at 72 °C for 30 s, a total of 40 cycles. There are three complex pores in all reactions. U6 was used as an internal reference for miR-150-5p expression, while glyceraldehyde-3-phosphate dehydrogenase (GAPDH) was used for SLCO4A1-AS1 and SLCO4A1. The ratio of the expression of the target gene in the experimental group and the control group was indicated as 2^−△Ct.^ △Ct = Ct (target gene) − Ct (internal reference) [13]. The primers (Table 1) of SLCO4A1-AS1, miR-150-3p and SLCO4A1 were synthesized by Sangon Biotech Co., Ltd. (Shanghai, China).

**Table 1:** Primer sequence for RT-qPCR.

Gene	Sequence (5′–3′)
U6	F: 5′-CTCGCTTCGGCAGCACA-3
	R: 5′-AACGCTTCACGAATTTGCGT-3′
GAPDH	F: 5′-GGAGCGAGATCCCTCCAAAAT-3′
	R: 5′-GGCTGTTGTCATACTTCTCATGG-3′
SLCO4A1	F: 5′-CTGCTCGCCCGTCTACATTG-3′
	R: 5′-CCGAGGGTAACAAGGATCG-3′
miR-150-3p	F: 5′-TCTCCCAACCCTTGTACCAGTG-3′
	R: 5′-CAGTGCGTGTCGTGGAGT-3′
LncRNA SLCO4A1-AS1	F: 5′-AGCATGTCCCACAAAGAGGG-3′

*F* forward, *R* reverse, *RT-qPCR* reverse transcription quantitative polymerase chain reaction, *GAPDH* glyceraldehyde-3-phosphate dehydrogenase, *SLCO4Al* solute carrier organic anion transporter family member 4A1, *miR-150-3p* microRNA-150-3p, *lncRNA SLCO4A1-AS1* long non-coding RNA SLCO4A1 antisense RNA 1.

### Western blot analysis

Rhe cells were lysed with radioimmunoprecipitation lysis buffer on ice for 30 min 48 h after transfection, centrifuged at 14,000 × *g* for 10 min, and then supernatants were collected. The protein concentration was determined by bicinchoninic acid protein assay kit. Proteins were separated with 10% sodium dodecyl sulfate-polyacrylamide gel electrophoresis and transferred onto the nitrocellulose membranes using a wet transfer method. The membrane was then blocked with 5% bovine serum albumin (BSA) for 1 h at room temperature and incubated overnight at 4 °C with the diluted rabbit polyclonal primary antibody to SLCO4A1 (ab122123, 1: 500) and GAPDH (ab8245, 1: 500). Above antibodies were purchased from Abcam Inc., (Cambridge, MA, USA). The membrane was then washed 3 times with Phosphate-Buffered Saline/Tween (PBST) and incubated with the horseradish peroxidase labeled goat anti-rabbit immunoglobulin G (IgG) secondary antibody (1:2000, ab6721, Abcam Inc., Cambridge, MA, USA) at room temperature for 1 h. Subsequently, the membrane was washed again with PBST for three times (15 min/time) and developed using diaminobenzidine. Images were captured using the Gel imager (Gel Doc XR, Bio-Rad, Hercules, CA, USA). The ratio of band intensity of the target protein to that of the internal reference was indicated as the relative expression of the gene.

### Fluorescence in situ hybridization (FISH)

The subcellular localization of SLCO4A1-AS1 in CD133^+^CD44^+^ colon cancer cells was detected using the FISH Kit (C10910, Guangzhou RiboBio Co., Ltd., Guangzhou, Guangdong, China). HCT116 cells in logarithmic growth phase were transferred onto slides in a 24-well plate (about 6 × 10^4^ cells/well). When cell confluence reached ~60–70%, the cells were fixed with 4% paraformaldehyde at room temperature for 10 min, and permeabilized with 1 mL pre-cooled Triton X-100 at 4 °C for 5 min. Cells were blocked with 200 μL pre-hybrid solution at 37 °C for 30 min. After that the cells were incubated with 2.5 μL hybridization solution containing 20 μM FISH Probe Mix overnight at 37 °C in dark, washed with solution I, solution II and solution III respectively at 42 °C. The cells were washed with 1× PBS for 5 min at room temperature. Next, cells were stained using 6-diamidino-2-phenylindole for 10 min and washed in 1× PBS for three times (5 min each time), after which the cells mounted and observed. The specific probe for lncRNA SLCO4A1-AS1 was synthesized by Guangzhou RiboBio Co., Ltd. (Guangzhou, Guangdong, China).

### RNA pull-down

CD133^+^CD44^+^ cells were transfected with 50 nM biotinylated WT-bio-miR-150-3p and Mut-bio-miR-150-3p. Cells were collected and washed with PBS 48 h after transfection. The cells were incubated in specific lysis buffer (Ambion, Austin, Texas, USA) for 10 min, centrifuged at 14,000 × *g* and the supernatants collected. The cell lysis was incubated with M-280 streptavidin magnetic beads (S3762, Sigma-Aldrich Chemical Company, St Louis, MO, USA) pre-coated with RNase-free BSA and yeast tRNA (TRNABAK-RO, Sigma-Aldrich Chemical Company, St Louis, MO, USA) for 3 h at 4 °C, and washed twice with pre-cooled lysis buffer, three times with low-salt buffer, and once with high-salt buffer. The bound RNA was purified by Trizol, and the expression of SLCO4A1-AS1 was determined by RT-qPCR.

### RNA immunoprecipitation (RIP)

HCT116 cells were lysed with lysis buffer (25 mM Tris-HCl, pH = 7.4; 150 mM NaCl, 0.5% NP-40; 2 mM EDTA; 1 mM NaF; 0.5 mM Dithiothreitol) containing a mixture of RNasin (Takara Holdings Inc., Kyoto, Japan) and protease inhibitor (B14001a, Roche, Basel, Switzerland) and centrifuged at 12,000 × *g* for 30 min followed by the collection of supernatants. The cell lysate was then incubated with anti-human EpCAM magnetic beads (130-061-101, Shanghai univ-bio, Co., Ltd., Shanghai, China) for 4 h at 4 °C. Then the beads were washed three times with wash buffer (50 mM Tris-HCl; 300 mM NaCl pH = 7.4; 1 mM MgCl 2; 0.1% NP-40). RNA was extracted from the magnetic beads using Trizol, and SLCO4A1-AS1 expression was determined by RT-qPCR.

### Xenograft tumor in nude mice

A total of 80 BALB/c specific-pathogen-free nude mice (age: 4 weeks, weight: 18–25 g; regardless of sex) were purchased from Hunan SJA Laboratory Animal Co., Ltd. (Hunan, China). A total of 1 × 10^6^ CD133^+^CD44^+^-HCT116 cells stably transfected with 8 μL vector SLCO4A1, si-SLCO4A1, miR-150-3p mimic, miR-150-3p inhibitor or oe-SLCO4A1-AS1 were inoculated subcutaneously into the nude mice. Tumor volume was monitored once a week. Tumor volume (V) was calculated using the formula: V = π/6 (height × length × width). Finally, the nude mice were euthanized at the end of the third week and the tumors were removed for analysis.

### Statistical analysis

All data were analyzed by SPSS 21.0 software (IBM Corp. Armonk, NY, USA). Measurement data were expressed as mean ± standard deviation. Unpaired data obeying normal distribution and homogeneity of variance between two groups were compared using unpaired *t*-test. Data among multiple groups were analyzed by one-way analysis of variance (ANOVA), with Tukey’s post hoc test. Repeated measurement ANOVA was used to analyze the data at different time points during the experiment, followed by Bonferroni’s post hoc test. A value of *p* < 0.05 was considered to be statistically significant.

## Results

### SLCO4A1 is highly expressed in colon cancer and correlates with high expression of SLCO4A1-AS1

**Fig. 1:**
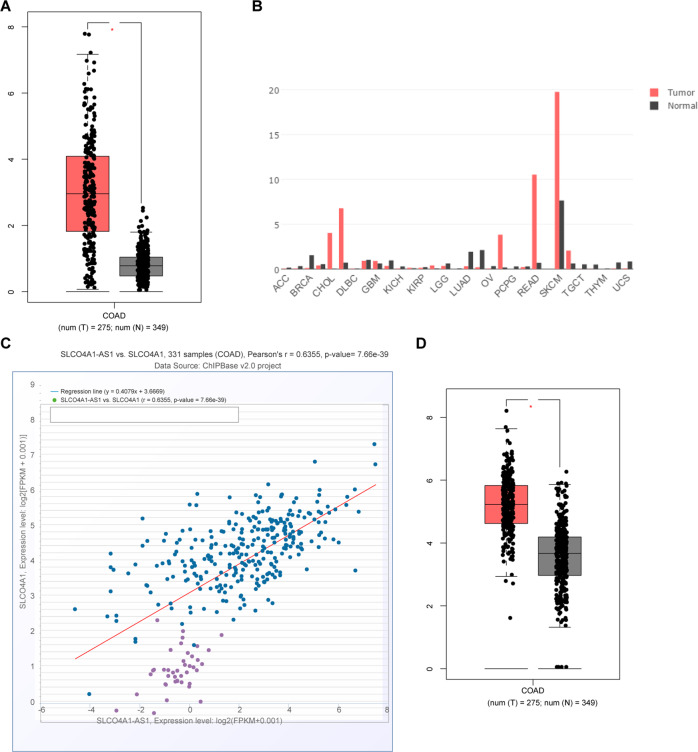
High expression of SLCO4A1 and SLCO4A1-AS1 are found in colon cancer. **A** Expression of SLCO4A1-AS1 in colon cancer tissues and adjacent normal issues based on GEPIA website. **B** Expression of SLCO4A1-AS1 in various tumor tissues and corresponding adjacent normal tissues retrieved through the GEPIA website. **C** Correlation analysis of expression of SLCO4A1-AS1 and SLCO4A1 based on the ChIPBase v2.0 website. **D** Expression of SLCO4A1 in colon cancer tissues and adjacent normal tissues retrieved using the GEPIA website.

Initially, microarray-based analysis was employed to screen the differentially expressed lncRNAs and mRNAs related to colon cancer. SLCO4A1-AS1 was found to be highly expressed in a variety of tumor types including colon cancer based on the GEPIA website (Fig. 1A, B). A positive correlation between the expression of SLCO4A1-AS1 and SLCO4A1 was found in colon cancer through the ChIPBase v2.0 website (Fig. 1C). Moreover, SLCO4A1 was also highly expressed in colon cancer through the GEPIA website (Fig. 1D). All in all, colon cancer exhibits the elevated expression of SLCO4A1-AS1 and SLCO4A1.

### SLCO4A1 is highly expressed in CD133+CD44+cells

**Fig. 2:**
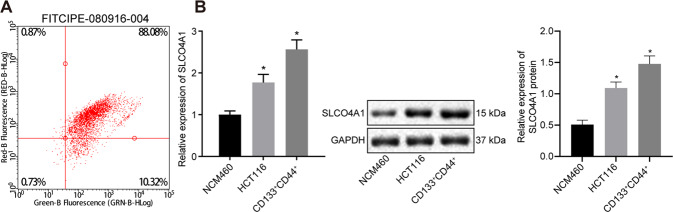
Elevated expression of SLCO4A1 is detected in CD133^+^CD44^+^ cells. **A** Expression of tumor stem cell maker CD133^+^CD44^+^ in human colon cancer HCT116 cells detected by flow cytometry. **B** Expression of SLCO4A1 in colon epithelial cells NCM460, HCT116 and CD133^+^CD44^+^ cells determined by RT-qPCR, expression of SLCO4A1 normalized to GAPDH, *p < 0.05 vs. colon epithelial cells NCM460, **p < 0.01 vs. HCT116 cells. The data were the measurement data and expressed as mean ± standard deviation. One-way ANOVA was used for the data analysis among multiple groups, followed by Tukey’s post hoc test. The cell experiment was repeated three times.

Next, the expression of SLCO4A1 in colon cancer cells was determined. Flow cytometry revealed that more than 90% of HCT116 cells were CD133^+^CD44^+^ × cells (Fig. 2A). Therefore, CD133^+^CD44^+^ × cells were selected for subsequent analysis. SLCO4A1 expression in this population was determined by RT-qPCR and western blot, which showed that SLCO4A1 expression in HCT116 cells was higher than in colon epithelial cells NCM460, while the highest expression of SLCO4A1 was found in CD133^+^CD44^+^ × cells (*p* < 0.05) (Fig. 2B). In a word, high expression of SLCO4A1 is detected in CD133^+^CD44^+^ × cells.

### Downregulation of SLCO4A1 inhibits the migration, invasion, sphere formation and tumor formation while promoting apoptosis of colon cancer stem cells

**Fig. 3:**
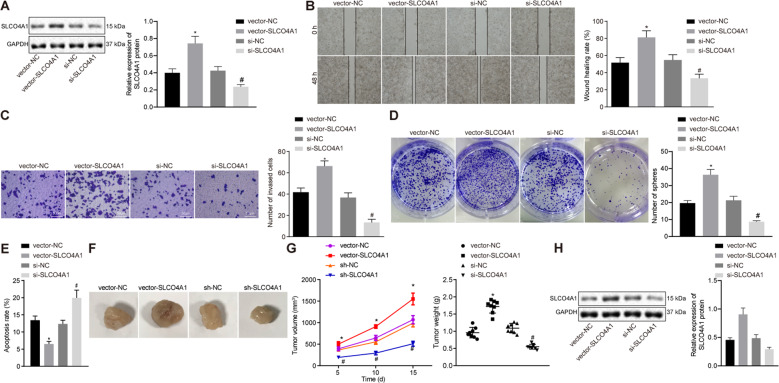
Knockdown of SLCO4A1 reduces migration, invasion, sphere formation and tumorigenesis abilities but enhances the apoptosis of colon cancer stem cells. **A** SLCO4A1 expression examined by western blot. **B** The migration, at 0 and 48 h, ability of CD133^+^CD44^+^ cells detected by scratch test. **C** The invasion ability of CD133^+^CD44^+^ cells detected by transwell assay. **D** Sphere formation ability of CD133^+^CD44^+^ cells assessed by suspension sphere formation assay. **E** The apoptosis of CD133^+^CD44^+^ cells detected by flow cytometry. **F** Weight of the tumors in nude mice. **G** The volume of the tumor in nude mice. **H** SLCO4A1 expression in tumors of nude mice examined by western blot. *p < 0.05 vs. cells treated with vector NC, ^#^*p* < 0.05 vs. cells transfected with si-NC. Data were measurement data and expressed as mean ± standard deviation. Data between two groups were analyzed by unpaired *t*-test and data among multiple groups at different time points were analyzed using repeated measurement ANOVA, together with Bonferroni’s post hoc test. *n* = 8. The cell experiment was repeated three times.

To determine the effects of SLCO4A1 has on colon cancer CD133^+^CD44^+^ × cells, we upregulated or knocked down the expression level of SLCO4A1 in CD133^+^CD44^+^-HCT116 cells, and the transfection effect was detected by western blot (Fig. 3A). Scratch test and transwell assay indicated that the migration and invasion abilities of these cells were significantly reduced by knockdown of SLCO4A1 (*p* < 0.05, Fig. 3B, C). In addition, the sphere formation ability of cells was also significantly decreased after silencing SLCO4A1 (Fig. 3D). Moreover, flow cytometry analysis revealed that the rate of cancer cell apoptosis was significantly increased in cells transfected with si-SLCO4A1 (*p* < 0.05, Fig. 3E). Therefore, downregulating SLCO4A1 appears to suppress the migration, invasion, sphere formation and accelerate apoptosis of colon cancer stem cells. These findings were also examined in vivo through xenograft transplantation in nude mice. The weight and volume of tumors in mice injected with si-SLCO4A1-treated cells were significantly lower than the mice injected with the si-NC-treated cells (*p* < 0.05) (Fig. 3F, G). Results were also validated at the protein level (Fig. 3H). Therefore, silencing SLCO4A1 expression inhibits tumorigenesis of colon cancer stem cells.

### miR-150-3p directly targets SLCO4A1

**Fig. 4:**
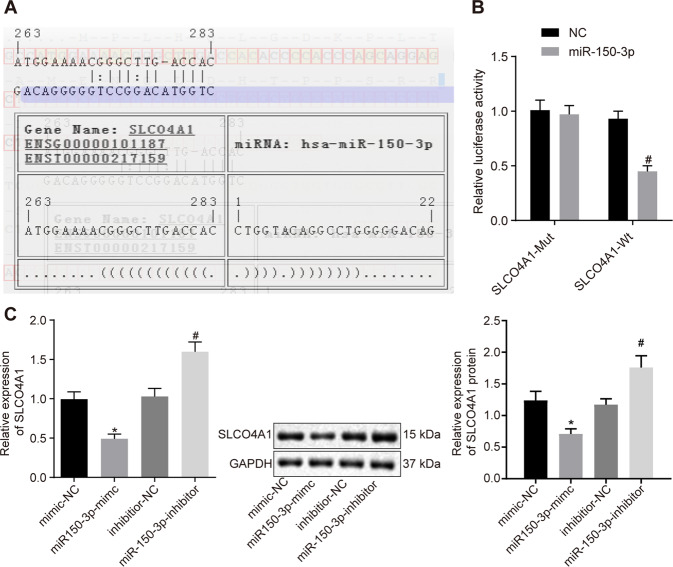
miR-150-3p specifically binds to SLCO4A1. **A** Verification of the binding site between miR-150-3p and SLCO4A1 using the online prediction software. **B** Relationship between miR-150-3p and SLCO4A1 verified by the dual-luciferase reporter assay. *p < 0.05 vs. NC treatment. **C** Expression of SLCO4A1 in CD133^+^CD44^+^ cells determined by RT-qPCR and western blot analysis, expression of SLCO4A1 normalized to GAPDH. *p < 0.05 vs. cells treated with mimic NC, ^#^*p* < 0.05 vs. cells treated with inhibitor NC. Data were measurement data and expressed as mean ± standard deviation. Unpaired *t*-test was used for the comparison between two groups. The experiment was repeated three times.

Next, we aimed to determine the relationship between miR-150-3p and SLCO4A1. Using online prediction software, we identified a binding site of miR-150-3p on the 3′UTR of SLCO4A1 (Fig. 4A). Dual-luciferase reporter assay showed that the luciferase activity of SLCO4A1-Wt 3′UTR was reduced by miR-150-3p relative to mimic NC (*p* < 0.05), while the luciferase activity of SLCO4A1-Mut was not inhibited by miR-150-3p (*p* > 0.05, Fig. 4B). These results indicate that miR-150-3p can specifically target SLCO4A1 3′UTR. Concurrently, RT-qPCR and western blot analysis demonstrated that the expression of SLCO4A1 in response to miR-150-3p mimic was significantly decreased relative to treatment with mimic NC (*p* < 0.05). In addition, miR-150-3p inhibitor led to the elevated SLCO4A1 expression (*p* < 0.05, Fig. 4C). These results indicate that SLCO4A1 is a target gene of miR-150-3p.

### miR-150-3p-mediated downregulation of SLCO4A1 inhibits migration, invasion, sphere formation, tumorigenesis and enhances apoptosis of colon cancer stem cells

**Fig. 5:**
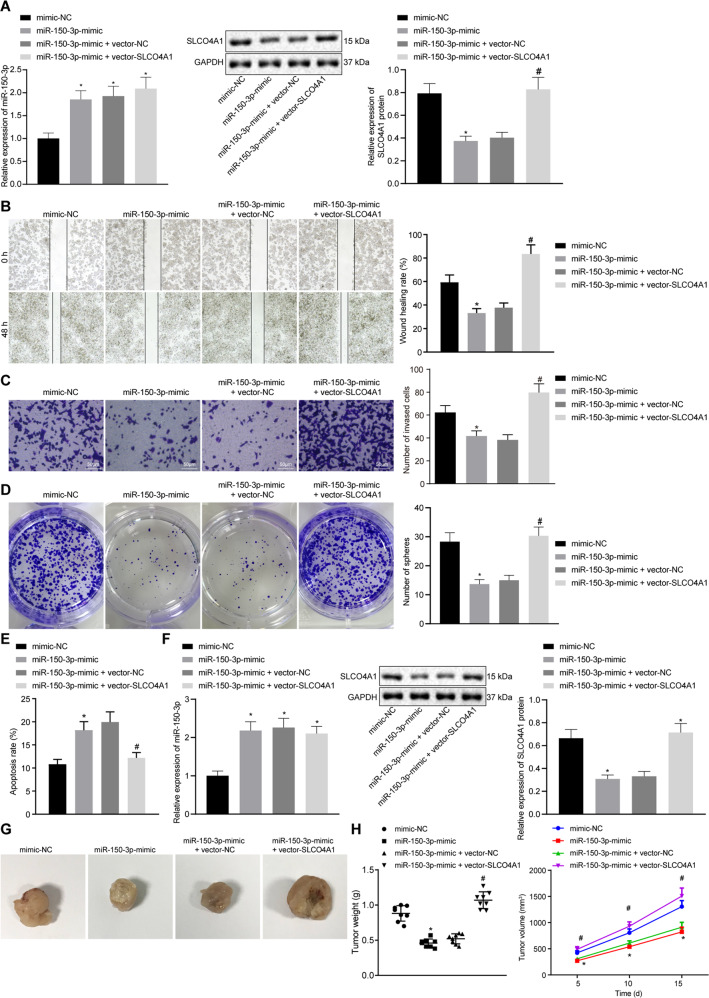
Overexpression of miR-150-3p downregulates SLCO4A1, resulting in repressed migration, invasion, sphere formation and tumorigenesis but potentiated apoptosis of colon cancer stem cells. **A** SLCO4A1 expression in CD133^+^CD44^+^-HCT116 cells detected by western blot; **B** migration ability of CD133^+^CD44^+^ cells detected by scratch test. **C** Invasion ability of CD133^+^CD44^+^ cells detected by transwell assay. **D** Sphere formation ability of CD133^+^CD44^+^ cells measured by suspension sphere formation assay. **E** Apoptosis of CD133^+^CD44^+^ cells detected by flow cytometry following Annexin V and PI staining. **F** miR-150-3p expression detected by qPCR; SLCO4A1 expression determined by western blot. **G** Tumor weight of nude mice. **H** Tumor volume of nude mice. *p < 0.05 vs. cells transfected with mimic NC, ^#^*p* < 0.05 vs. cells co-transfected with miR-150-3p mimic and vector NC. Data were measurement data and expressed as mean ± standard deviation. Unpaired *t*-test was used for the comparison of data between two groups. Repeated measurement ANOVA was applied for data comparison among multiple groups at different time points with Bonferroni’s post hoc test. *n* = 8.

To explore the effect of miR-150-3p on the development of colon cancer, we first used qPCR to verify the expression of miR-150-3p. Compared with the miR-NC, the expression of miR-150-3p was increased in response to miR-150-3p mimic, miR-150-3p mimic + vector NC or miR-150-3p mimic + vector SLCO4A1. The expression of SLCO4A1 was detected by western blot analysis. The results showed that, compared with the mimic NC, the expression of SLCO4A1 was decreased in response to miR-150-3p mimic. Compared with the miR-150-3p mimic + vector NC, the SLCO4A1 expression in the cells treated with miR-150-3p mimic + vector SLCO4A1 was increased (Fig. 5A). Scratch test and transwell assay revealed that the migration and invasion abilities were attenuated by miR-150-3p mimic treatment, which could be rescued by co-treatment of miR-150-3p mimic and oe-SLCO4A1 (*p* < 0.05, Fig. 5B, C). Suspension sphere formation assay showed that the cell sphere formation ability was significantly reduced in cells transfected with miR-150-3p mimic, which was blocked by co-transfection of miR-150-3p mimic and oe-SLCO4A1 (Fig. 5D). In addition, flow cytometry analysis indicated that the rate of apoptosis increased significantly following treatment with miR-150-3p mimic treatment, which was reversed by co-treated with miR-150-3p mimic and oe-SLCO4A1 (*p* < 0.05, Fig. 5E). The expression of miR-150-3p were increased in response to miR-150-3p mimic, miR-150-3p mimic + vector NC or miR-150-3p mimic + vector SLCO4A1 compared with the mimic NC. The expression level of SLCO4A1 in mice treated with miR-150-3p mimic was significantly lower than that in miR-150-3p mimic, and SLCO4A1 expression in mice treated with miR-150-3p mimic + vector NC was higher than that in miR-150-3p mimic + vector SLCO4A1 (Fig. 5F). Moreover, the xenograft transplantation in nude mice showed that tumor weight and volume were significantly lowered in mice introduced with miR-150-3p mimic-treated cells compared with the mice manipulated with mimic NC-treated cells (*p* < 0.05). Tumor weight and volume of mice in response to co-treatment of miR-150-3p mimic and oe-SLCO4A1 were notably increased compared with the mice in response to co-treatment of miR-150-3p mimic and oe-NC (*p* < 0.05, Fig. 5G, H). These findings provide evidence demonstrating that the overexpression of miR-150-3p downregulated SLCO4A1 and that this process inhibited the tumorigenesis of colon cancer stem cells in vivo.

### SLCO4A1-AS1 competitively binds to miR-150-3p leading to the increased levels of SLCO4A1 expression

**Fig. 6:**
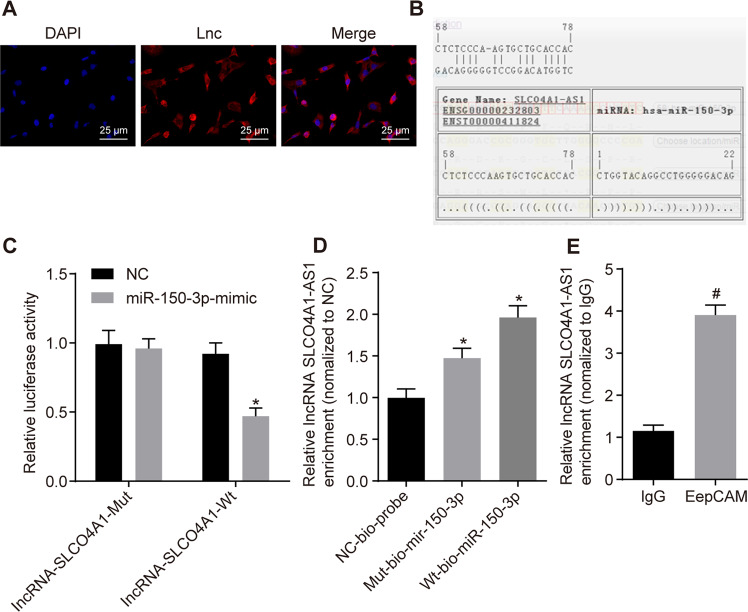
SLCO4A1-AS1 downregulates the expression of miR-150-3p, elevating the expression of SLCO4A1. **A** The localization of SLCO4A1-AS1A in cells detected by FISH. **B** The binding site between miR-150-3p and SLCO4A1-AS1 predicted using the online prediction software. **C** The relationship between miR-150-3p and SLCO4A1-AS1 detected by dual-luciferase reporter assay. *p < 0.05 vs. NC treatment. **D** Relative enrichment of SLCO4A1-AS1 by miR-150-3p detected by RNA pull-down. *p < 0.05 vs. Mut-bio-miR-150-3p. **E** SLCO4A1-AS1 and miR-150-3p binding to Ago2 protein determined by RIP. ^#^*p* < 0.05 vs. IgG. The data were the measurement data and expressed as the mean ± standard deviation. Unpaired *t*-test was applied for the comparison between two groups while one-way ANOVA was used among multiple groups, followed by Tukey’s post hoc test. The cell experiment was repeated three times.

Next, the intracellular interaction of SLCO4A1-AS1, miR-150-3p and SLCO4A1 were examined. FISH revealed that SLCO4A1-AS1 was mainly expressed in the cytoplasm of CD133^+^CD44^+^ cells, suggesting that its main function may be in the cytoplasm (Fig. 6A). Online prediction software indicated that there was a miR-150-3p binding site in SLCO4A1-AS1 which could facilitate this interaction (Fig. 6B). This finding was investigated in vitro using a dual-luciferase reporter assay. The luciferase activity of SLCO4A1-AS1-Wt was clearly inhibited by miR-150-3p, while that of SLCO4A1-AS1-Mut was not affected, indicating that miR-150-3p can specifically bind to SLCO4A1-AS1 (Fig. 6C). An RNA pull-down assay displayed that the level of SLCO4A1-AS1 bound by WT-miR-150-3p was significantly increased compared with Mut-miR-150-3p (*p* < 0.05, Fig. 6D), suggesting that miR-150-3p can directly bind to SLCO4A1-AS1. Next, RIP assay showed that the expression of Argonaute 2 (Ago2)-bound SLCO4A1-AS1 was significantly increased compared to IgG-bound SLCO4A1-AS1 (*p* < 0.05, Fig. 6E), indicating that SLCO4A1-AS1 can also bind to Ago2 protein. These results suggest that SLCO4A1-AS1 may participate in the regulation of SLCO4A1 by competitively binding to miR-150-3p.

### SLCO4A1-AS1 accelerates colon cancer progression by binding to miR-150-3p and upregulating SLC04A1

**Fig. 7:**
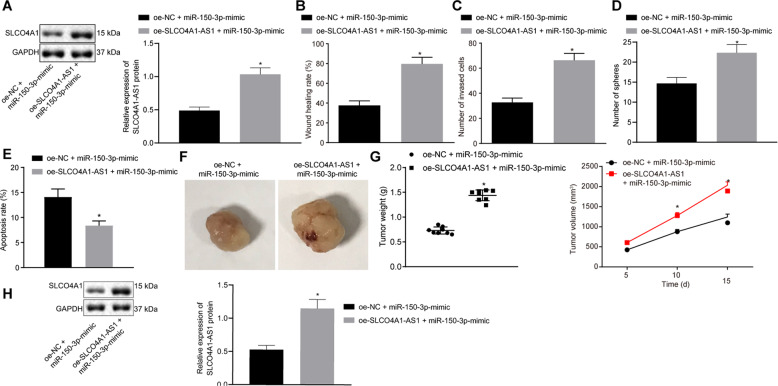
SLCO4A1-AS1 augments migration, invasion, sphere formation and tumorigenesis abilities but inhibits apoptosis of colon cancer stem cells by binding to miR-150-3p. **A** SLCO4A1-AS1 expression in CD133^+^CD44^+^ cells and HCT116 cells detected by western blot assay. **B** The migration ability of CD133^+^CD44^+^ cells detected by scratch test. **C** The invasion ability of CD133^+^CD44^+^ cells detected by transwell assay. **D** The sphere formation ability of CD133^+^CD44^+^ cells detected by suspension sphere formation assay. **E** The apoptosis of colon cancer cell CD133^+^CD44^+^ cells detected by flow cytometry. **F** The weight of tumors of nude mice. **G** The volume of tumors of nude mice. **H** SLCO4A1-AS1 expression in tumors of nude mice detected by western blot assay. *p < 0.05 vs. cells co-transfected with oe-NC and miR-150-3p mimic. The data were the measurement data and expressed as the mean ± standard deviation. Unpaired *t*-test was applied for the comparison of data between two groups. The cell experiment was repeated three times. Repeated measurement ANOVA was applied for data comparison between groups at different time points with Bonferroni’s post hoc test. *n* = 8. The cell experiment was repeated three times.

To explore the effect of SLCO4A1-AS1 binding to miR-150-3p on the development of colon cancer, the expression of SLCO4A1-AS1 protein was detected by Western Blot. Results showed that SLCO4A1-AS1 expression was increased in cells co-treated with oe-SLCO4A1-AS1 and miR-150-3p mimic compared with the cells co-treated with oe-NC and miR-150-3p mimic (*p* < 0.05, Fig. 7A). Scratch test and transwell assay suggested that the migration and invasion abilities of the cells co-treated with oe-SLCO4A1-AS1 and miR-150-3p mimic were significantly enhanced compared with the cells co-treated with oe-NC and miR-150-3p mimic (*p* < 0.05, Fig. 7B, C). According to suspension sphere formation assay, the sphere formation ability of the cells co-transfected with oe-SLCO4A1-AS1 and miR-150-3p mimic was significantly enhanced versus the cells co-treated with oe-NC and miR-150-3p mimic (Fig. 7D). As shown previously, flow cytometry analysis showed that the apoptosis rate was significantly lowered in cancer cells in response to co-treatment of oe-SLCO4A1-AS1 and miR-150-3p mimic versus co-treatment of oe-NC and miR-150-3p mimic (*p* < 0.05, Fig. 7E). In addition, the xenograft tumor in nude mice displayed increased tumor weight and volume in mice injected with of cells co-treated with oe-SLCO4A1-AS1 and miR-150-3p mimic (*p* < 0.05, Fig. 7F, G). Results were also validated at the protein level (Fig. 7H). In summary, we can preliminarily draw the following conclusions: SLCO4A1-AS1 binds to miR-150-3p to promote SLCO4A1 expression and thus promote the characteristics of colon cancer stem cells

## Discussion

Although a significant number of researches is underway attempting to identify pathologic, molecular prognostic and immunologic targets for colon cancer [14], the underlying molecular mechanism of colon cancer progression remains poorly understood. Previous reports have identified a small number of lncRNAs which are abnormally expressed in colon cancer [15]. These non-coding RNAs are of great importance in the prevention, diagnosis and treatment of patients with colon cancer. However, it remains unclear how SLCO4A1-AS1 affects colon cancer progression. Here, we explored the role of the lncRNA SLCO4A1-AS1 in colon cancer stem cells. We demonstrated that SLCO4A1-AS1 knockdown downregulated the expression of SLCO4A1 and thus inhibited the development of colon cancer stem cells by relieving the inhibition on miR-150-3p.

Our study demonstrated that SLCO4A1 was highly expressed in colon cancer cells and promoted their proliferation. A recent study demonstrated that SLCO4A1 was highly expressed in several other cancers including colorectal cancer, and it served as a prognostic marker for colorectal cancer [9]. In addition, the expression of SLCO4A1 is strongly increased in ovarian cancers [16]. Interestingly, organic anion transporting polypeptides, uptake transporters of the solute carrier family, are found to be elevated in diverse cancers, such as colorectal cancer and pancreatic cancer [17, 18]. Moreover, the study showed that SLCO4A1 was downregulated by miR-150-3p, which can inhibit colon cancer stem cell progression. Recently, a study displayed that miR-150-3p downregulated the expression of transcription factor Specificity Protein 1, thus attenuating the proliferation ability and growth of glioma cell [19]. miR-150-3p could function as a tumor-suppressor in both head and neck squamous cell carcinoma and the overexpression of miR-150-3p bound to SPOCK1 to alleviate the aggressiveness of head and neck squamous cell carcinoma [20]. Similarly, expression of TNS4 was downregulated by miR-150-3p to suppress lung adenocarcinoma cell development [21].

The data in this study build on these results, confirming that SLCO4A1-AS1 was highly expressed in colon cancer tissues, and downregulation of SLCO4A1-AS1 could exert an inhibitory function over the development of colon cancer. Concurrently, SLCO4A1-AS1 was revealed to be elevated in the tissues of colorectal cancer, which served as a predictor of poor prognosis and metastasis of colorectal cancer [22]. These results fit with a recent study which showed that the expression of SLCO4A1-AS1 was upregulated in bladder cancer, and knockdown of SLCO4A1-AS1 was able to inhibit the cellular progression of bladder cancer [7].

Our data demonstrated that the downregulation of SLCO4A1-AS1 increased miR-150-3p to downregulate SLCO4A1 expression, thus inhibiting the development of colon cancer stem cells. Recently, lncRNAs have been implicated to regulate the expression and function of miRNAs, thus participating in disease development [23]. For instance, an investigation exhibited that lncRNA LOXL1-AS1 downregulation inhibited prostate cancer progression through upregulating miR-541-3p and reducing CCND1 [24]. Interestingly, another study provided evidence that lncRNA RP4 competitively bound to miR-7-5p to elevate the expression of SH3GLB1 gene, thus repressing the biological processes of colorectal cancer [25]. Similarly, downregulating lncRNA-HEIH protects against colorectal cancer tumorigenesis by increasing the expression of miR-939, mediating transcriptional repression of Bcl-xL [26]. Those findings supported the conception that knocking down SLCO4A1-AS1 was able to inhibit colon cancer progression by upregulating miR-150-3p to downregulate SLCO4A1.

**Fig. 8:**
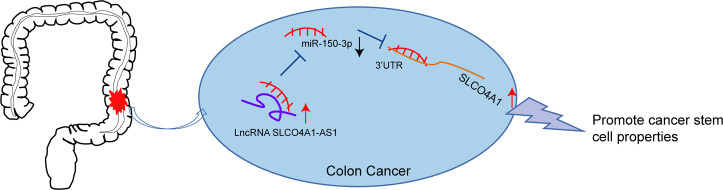
The potential role of LncRNA SLCO4A1-AS1 in the progression of colon cancer by regulating microRNA-150-3p/SLCO4A1 axis. SLCO4A1-AS1 inhibits SLCO4A1-AS1 knockdown downregulated expression of SLCO4A1 to suppress the development of colon cancer stem cells by relieving the inhibition on miR-150-3p.

In summary, the data presented here showed that the downregulation of SLCO4A1-AS1 inhibited SLCO4A1 expression to protect against colon cancer progression by competitively binding to miR-150-3p (Fig. 8). Therefore, SLCO4A1-AS1 may be a new biomarker for colon cancer, a marker for patient prognosis and a promising therapeutic target for colon cancer treatment.

## Data Availability

The datasets used and analyzed in the current study are available from the corresponding author upon request.
